# The High-Flatness Mounting Process of Image Sensor Splicing Units with Ultra-Large Format Focal Plane

**DOI:** 10.3390/s26216783

**Published:** 2026-10-27

**Authors:** Hongjian Li, Linxiao Jing, Lipeng Jing, Haoran Li, Qijun Zhou, Yongchao Wu

**Affiliations:** Beijing Microelectronic Technology Institute, Beijing 100076, China; lihongjian341@163.com (H.L.); jinglx0330@163.com (L.J.); jlpeng110@163.com (L.J.); haoran1997@tju.edu.cn (H.L.); schaffhausen61689@163.com (Q.Z.)

**Keywords:** image sensor, ultra-large-format focal plane, chip splicing, flatness, reliability

## Abstract

Image sensor chip splicing technology plays a crucial role in enhancing the imaging capabilities of current ultra-large-format focal plane imaging systems for both space-based and ground-based ultra-large-format focal plane imaging systems, where the imaging performance heavily relies on the flatness of the chip mounted on the substrate within the splicing unit. Based on the analysis of the influencing factors of chip-mounting flatness, this research developed a high-flatness mounting methods for packaging structure of chip-adhesive-substrate, irrespective of focal plane size. Through the coordinated optimization of packaging materials, structural dimensions, and mounting processes, high-flatness mounting of image sensor chips can be achieved. The substrate material and thickness, along with chip thickness exert a more pronounced impact on chip-mounting flatness compared to mounting process variables like dispensing pattern, adhesive thickness, and curing curve. Furthermore, for substrate and chip with specific size, there exists an optimal chip thickness that yields the best flatness for chip mounting. Based on this method, this research successfully achieved superior flatness mounting of a 50 mm × 96 mm sized large-format focal plane on an AlN substrate by employing both large-sized single-chip mounting and multi-small-sized chip splicing mounting techniques. Specifically, the flatness of the large-sized single-chip mounting reached 2.8 μm, while the flatness of the 2 × 3 chip splicing array attained 11.6 μm; the flatness of these samples did not show significant change after consecutive tests of 100 h aging at 100 °C and 100 times thermal cycling (−55 °C to 100 °C).

## 1. Introduction

The growing demand for more expansive imaging ranges and enhanced resolution in space-based and ground-based ultra-large-format focal plane imaging systems, including remote sensing and mapping satellites, reconnaissance pods, and sky survey telescopes, necessitates the advancement of imaging systems to a couple billion, or even a hundred-billion, pixels [1]. This demand, in turn, propels the evolution of image sensor chips toward reducing pixel sizes and increasing imaging focal plane size [2,3]. Ensuring micron-level flatness of the ultra-large-format focal plane composed of image sensor chips is essential for simultaneous normal imaging of all pixels. The flatness error of the CCD focal plane must be rigorously confined within the depth of focus. Failure to do so will cause part of the target surface to deviate from the image plane of the optical system, resulting in inconsistent image sharpness across the field. Consequently, the flatness requirement for the ultra-large-format focal plane typically needs to reach 20–30 μm [4,5].

Ultra-large survey telescopes at home and abroad are all implemented by means of image sensor chip unit splicing, and splicing units are composed of large-sized single-chip mounting or small-sized multiple-chip-splicing mounting [6,7]. Ensuring the high-flatness focal plane of the splicing unit (approximately 10 μm) is an essential precondition for the high flatness of the ultra-large-format focal plane [8,9,10,11]. For splicing units fabricated by large-sized single chip, the mounting process is relatively straight forward. However, due to variations in material properties such as chip initial warpage, substrate roughness, coefficient of thermal expansion (CTE) and elastic modulus, it becomes more challenging for achieving micron-level mounting flatness as the chip size increases [12]. Additionally, the alternate temperature variations can induce thermal mismatch in the packaging system, resulting in flatness degradation that adversely affects imaging quality. In contrast, multiple-chip-splicing mounting allows for easier attainment of high flatness with smaller individual chips, but it cannot guarantee the splicing accuracy between chips and the overall flatness of the splicing units. Table 1 summarizes the flatness of ultra-large-format focal plane splicing systems both domestically and internationally, in which the mounting flatness of individual chip is consistently ≤20 μm [13,14,15,16,17,18,19,20,21,22].

Image sensor chips are extensively utilized across various domains, including mobile phones, cameras, aerospace, automotive electronics, and security. The packaging types encompass traditional ceramic packages [23,24], Chip on Board (COB) [25,26], Flip-chip, and the current advanced Wafer-Level Packaging (WLP) [27,28,29,30]. This research concentrates on high-reliability image sensors for aerospace applications and conducts process studies on the typical packaging structure of chip–adhesive–ceramic substrate.

**Figure 1:**
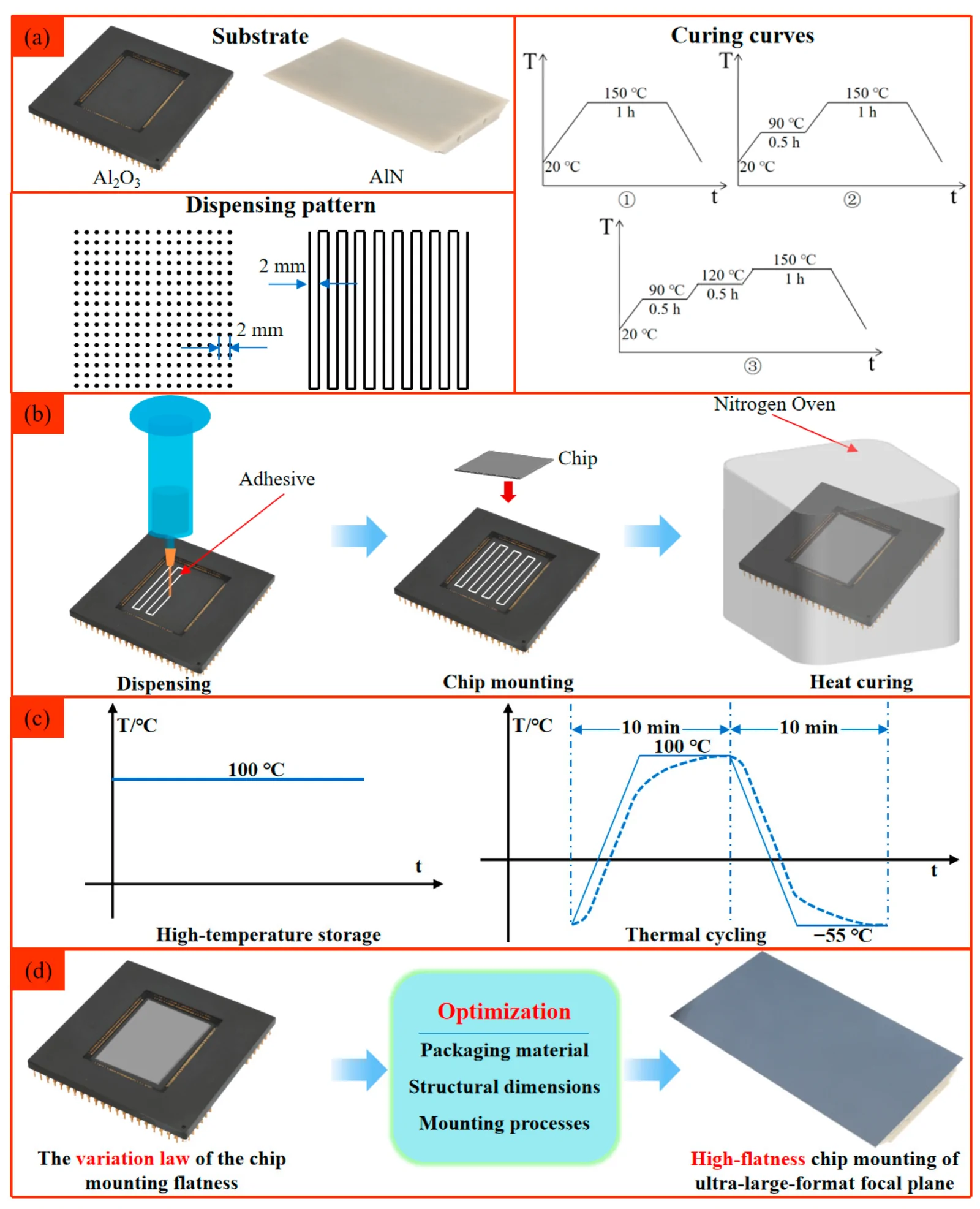
(**a**) Packaging processes; (**b**) adhesive bonding process; (**c**) temperature curve of reliability tests; and (**d**) research strategy of this study.

The adhesive bonding process is currently the predominant packaging method for large-sized image sensor chips. This study aims to achieve high-flatness mounting of image sensor chips for ultra-large-format focal plane splicing units measuring 50 mm × 96 mm. Initially, the influence laws of the mounting process (dispensing pattern, adhesive thickness and curing curve) and packaging material (substrate material, chip thickness) on the flatness of chip mounting were discussed through single-chip mounting tests and finite element simulations of the splicing unit. Subsequently, based on the influence law, this research successfully achieves high-flatness mounting for both the 50 mm × 96 mm sized single chip and 2 × 3 chip arrays (Figure 1d). The results of this research enhance the comprehension of flatness control in image sensor packaging and establish a foundation for the subsequent high-flatness splicing packaging of billion- or even hundred-billion-pixel format focal planes.

## 2. Methods

### 2.1. Experimental Materials and Equipment

In this study, silicon wafers of varying sizes and thicknesses were utilized as chips, which were subsequently attached to Al_2_O_3_ and AlN substrates using an adhesive (Figure 1a). Chips of the same thickness are produced by thinning and cutting from the same wafer. Al_2_O_3_ and AlN have emerged as the preferred materials for packaging image sensors due to their distinctive effective thermal management, thermal–mechanical compatibility, and radiation resistance. The Al_2_O_3_ substrate (bulk product supplied by our company) was specifically employed to attach 25 mm × 32 mm sized chips for investigating the variation law of chip-mounting flatness, while the AlN substrate was utilized for ultra-large-format focal plane splicing units, attaching a large-sized single chip or 2 × 3 chip arrays measuring 50 mm × 96 mm. The adhesive was dispensed on the substrate using a SHOTmini100SX dispensing machine (Tokyo, Japan), with the dispensing pattern illustrated in Figure 1a. Subsequently, the chip attachment was executed using the MRSI-705 high-precision mounting machine (Tewksbury, MA, USA), followed by curing in the ANO-640B nitrogen oven (Dongguan, China). Three curing curves are depicted in Figure 1a. To experimentally investigate the variation law of mounting flatness, three samples were tested for each parameter (the standard deviation of each parameter was calculated: error bar). The adhesive bonding process is illustrated in Figure 1b, including dispensing, chip mounting, and heat curing.

### 2.2. The Die Attachment of 2 × 3 Image Sensor Chip Array

Upon completion of the dispensing process on the AlN substrate, the chips of the 2 × 3 array were mounted in sequence. However, low flatness of the spliced unit occurred due to the height fluctuations among the chips in the array arising from mechanical errors of the chip mounter, the uniformity of the mounting pressure, and roughness deviations at different substrate positions, necessitating subsequent fine-tuning of the chip array to mitigate the initial misalignment induced by the mounting process. Finally, curing was performed according to curing curve ①.

Fine-tuning methods: Initially, the degree and direction of the warpage for each chip were assessed using X-ray scanning (Figure S1). Subsequently, the clarity of the connection points among 4 chips was examined with an optical microscope to evaluate the height of the chips (Figure S2). The taller chips were due to a tilt during the mounting process. For chips with a taller position, a gentle pressing action (using a lint-free clean swab) was applied to align the heights of the four chips. The gentle pressing action was about 10–20 g with a 1–2 s pressing time, and the pressing position of the taller chips was the region shown in Figure S2 (the corner of the chips). The edge of each chip typically has a region without pixels that can be pressed. The height of the microscope objective lens is adjusted by the fine focusing knob. The fine focusing knob has a travel of 100 μm per full rotation and 1 μm per graduation. The relative height can be determined by observing the change in the graduations when the adjacent chips are clear of the lens. The process of measuring heights and conducting pressing maneuvers at the connection points of the four chips was reiterated until the height differential was deemed to be within 10 μm or further leveling became unfeasible, prompting the cessation of the flatness adjustment. The fine-tuning process occurred promptly following the mounting of the chips at room temperature.

### 2.3. Reliability Test

This study conducted high-temperature storage (at 100 °C) and thermal cycling tests (−55 °C to 100 °C) to evaluate the reliability of the mounting samples with ultra-large-format focal plane (50 mm × 96 mm sized single chip and 2 × 3 chip arrays). The temperature curve is shown in Figure 1c. Finally, the flatness of all samples with the ultra-large-format focal plane was measured following the high-temperature storage and thermal cycling tests.

### 2.4. Flatness Measurement

A Dektak-XT step gauge (measurement accuracy: 0.01nm) by Bruker Company (Tucson, AZ, USA) was employed to assess the flatness of the silicon wafers post-curing, applying a probe contact force of 3 mg during scanning. The focal plane size of the splicing unit was 50 mm × 96 mm. Height measurements of each point were recorded every 5 μm along the X-axis, with a step length of 2 mm in the Y-axis. The scanning speed was set to 1 mm/s. The total scanning time varied with the measured area. For the 50 mm × 96 mm sample, the total duration of one full-area scan was approximately 45 min. For the 25 mm × 32 mm sample, the total duration of one full-area scan was about 8 min. All measurements were performed in ambient air at room temperature. The data were processed using the Dektak XT test software, which generates the surface profile and relevant height of the area scanned by the probe. After skew correction, a 3D map image and the flatness of the chip surface were obtained.

### 2.5. Finite Element Analysis of the Chip Curing Process

This work employed the Ansys Workbench finite element simulation software to simulate the deformation of the packaging system during cooling process after thermal curing, investigating the impact of substrate materials (Al_2_O_3_, AlN, SiC) on the flatness of chip mounting. Given that this structure is symmetrically distributed around the center, an equivalent model encompassing only 1/4 of the structure was established to streamline the simulation. The structural model comprises three components: the substrate, the bonding adhesive (full surface filling), and the chips.

The purpose of the simulation in this study is to investigate the influence of packaging substrate materials on chip warpage. However, the heating and curing process of the adhesive involves a complex physical and chemical reaction. The initiation temperature of the curing process and the extent of cross-linking reaction at various temperatures during heating are challenging to quantitatively simulate. Therefore, this study employed a simplified model: The system remains unwrapped during the temperature-rise stage (with the adhesive in a fluid state and components freely expanding) and the substrate remains dimensionally stable during the constant-temperature curing process; warpage only occurs during the cooling stage. Thus, the simulation focused solely on the cooling process of the packaging system, and the results can only qualitatively depict the impact of the substrate material type on the flatness of the chip mounting. There is no relative sliding between the various structures. Therefore, bonded constraints (Bonded Contact) were implemented between the substrate and the adhesive film, as well as between the adhesive film and the chip.

**Figure 2:**
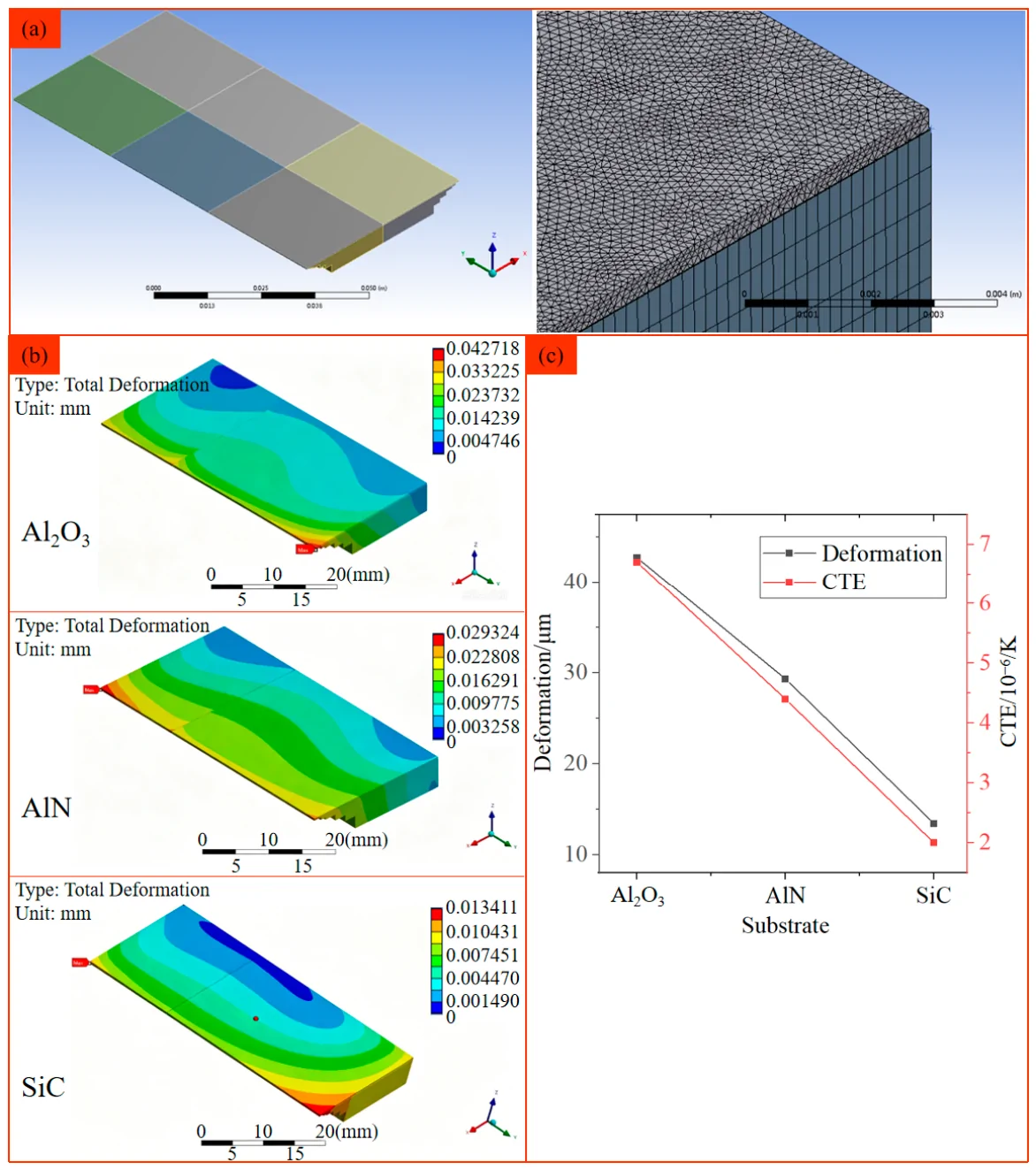
The flatness simulation results of the 2 × 3 image sensor splicing unit with different substrates. (**a**) The overall modeling structure and the mesh of the structure; (**b**) simulation result; (**c**) and the variation trend of the simulated deformation results and the changes in substrate CTE.

Figure 2 depicts the overall modeling structure, the structural diagram post meshing, and quarter-symmetric simulation model, all of which are grounded in the actual structure of a 2 × 3 image sensor splicing unit. The intrinsic characteristic parameters of the relevant materials are presented in Table 2. The chip dimensions were 25 mm × 32 mm, with a thickness of 400 μm. The substrate thickness measured 5 mm, and the surface mount dimensions were 47.5 mm × 96 mm. A fixed constraint was imposed on the bottom surface of the substrate at the outer corner points. Displacement constraints in the x and y directions were applied to two adjacent points at the center points of the outer edges. The mesh comprised hexahedral elements with element sizes of 0.2 mm for the substrate region, 0.02 mm for the adhesive, and 0.1 mm for the chip. The total number of structural nodes was 5,818,768, and the number of mesh elements was 3,874,422.

## 3. Results and Discussion

### 3.1. The Variation Law of the Chip-Mounting Flatness

This study utilized a combination of process tests and finite element simulations to investigate the impact of bonding process variables (dispensing pattern, adhesive layer thickness, curing curve) and packaging material characteristics (substrate material, chip thickness) on the flatness of single-chip mounting and chip-splicing mounting.

#### 3.1.1. Substrate Material

The inherent differences in materials properties such as coefficient of thermal expansion (CTE) and elastic modulus lead to varying degrees of CTE mismatch between different substrates and silicon (Si) chips. Packaging systems utilizing different substrates will experience varying thermal stresses and deformations during the cooling processes. This study conducted simulations to compare the impact of three substrate materials (Al_2_O_3_ ceramic, AlN ceramic, and SiC) on the flatness of chip array mounting. The results are presented in Figure 2b. The deformation varies significantly as the substrate material changes. The variation in flatness primarily stems from the differences in substrate CTE, as illustrated in Figure 2c. The trend of flatness alteration is consistent with that of substrate CTE. According to the materials parameters, it can be known that the SiC substrates possess the closest thermal expansion coefficients with Si chips, followed by AlN, while the variance between Al_2_O_3_ and Si chips is the most pronounced. Consequently, this leads to minimal stress generation during the curing process of the SiC-Si system, thereby reducing chip warpage.

Additionally, substrate thickness is also a critical factor that affecting the flatness of chip mounting. Jiang et al. [21] simulated the impact of substrate thickness on the CMOS chips warpage, revealing that as substrate thickness increased from 1 mm to 3.5 mm and chip warpage decreased from 8 μm to 3.5 μm. In this study, the material stiffness shows a direct proportionality to the cube of the material thickness. Consequently, a thicker substrate exhibits greater flexural strength and is less susceptible to bending deformation under equivalent stress. When the chip is mounted on the substrate, a thicker substrate more effectively restrains the chip’s deformation. Thus, increasing substrate thickness proves to be an effective strategy for enhancing the chip-mounting flatness.

Without considering other objective factors, the occurrence of chip warpage is attributed to temperature fluctuations during the curing process, as well as the mismatch in the CTE of the materials themselves. The warping process (for Al_2_O_3_ substrate) can be illustrated in Figure S3. During the heating process, the adhesive for the chip remains in a fluid state, allowing the chip and substrate to expand freely. After being held at 150 °C for 1 h, the adhesive solidifies, forming cross-linking among the chip, adhesive, and substrate. Upon cooling, the mismatch in CTE causes varying shrinkage in the volumes of the three components, resulting in stress accumulation. Consequently, the warpage of the chip progressively increases throughout the cooling process [31].

#### 3.1.2. Chip Thickness

**Figure 3:**
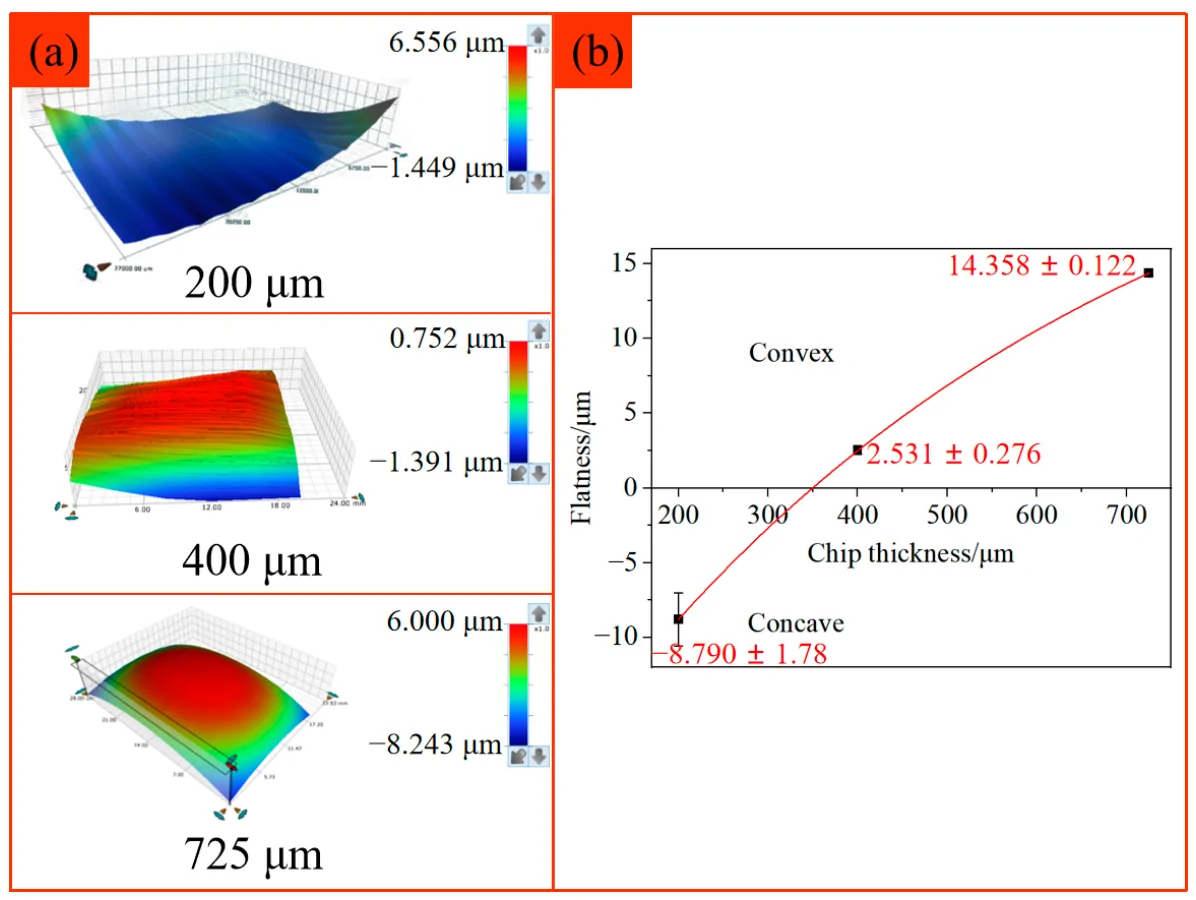
The trend of flatness variation between 25 mm × 32 mm size chips of different thicknesses. (**a**) Flatness measuring results and (**b**) trend of flatness change.

In this study, the Al_2_O_3_ substrate was utilized to experimentally assess the impact of chip thickness (200 μm, 400 μm, and 725 μm) on the flatness of 25 mm × 32 mm sized chip mounting. The dot matrix dispensing pattern and type ① curing curve were employed. The results are illustrated in Figure 3a. As the chip thickness increased, the warpage of the chip decreased from 8.8 μm in the concave shape to 2 μm in the convex shape, and then rose to 14 μm in the convex shape. The flatness alteration curve is depicted in Figure 3b. By observing the concave–convex transitions of the chip, it can be inferred that there is an optimal chip thickness theoretically capable of achieving minimum chip warpage.

Overall, the warpage trend of chips with varying thickness is primarily affected by two factors: the inherent warpage of Si chips and the deformation that occurs during the curing process. Si chips are produced by thinning and cutting Si wafers. For chips with an initial thickness of 725 µm that have not undergone thinning, the original warpage can be considered negligible, or effectively zero. In contrast, chips that have been thinned to thicknesses of 400 µm and 200 µm exhibit an initial warpage, as illustrated in Figure S4. The thinner the chip, the more pronounced the initial warpage resulting from the thinning process. During the curing process, the substrate experiences greater shrinkage deformation compared to the chip due to the difference in their coefficients of thermal expansion. For a chip with a thickness of 725 µm, the chip shrinks towards the center driven by the substrate and the adhesive. However, due to the obstruction of the substrate, the chip can only protrude upwards (variation: 14.358 µm). In the case of chips with thicknesses of 400 µm and 200 µm, upward convex deformation also occurs during curing under similar forces. The deformation of the 400 µm thick chip exceeds the warping of the chip, resulting in an overall upward convex morphology (variation: ~14.996 µm). Conversely, the deformation of the 200 µm thick chip is less than its inherent warping, which leads to an overall concave morphology (variation: ~16.541 µm). The variation in chip warpage suggests that as the chip becomes thinner, the impact of curing-induced deformation becomes more significant. This phenomenon can be attributed to the higher bending stiffness of thicker silicon chips, which suppresses the effects of curing-induced deformation.

#### 3.1.3. Dispensing Pattern

**Figure 4:**
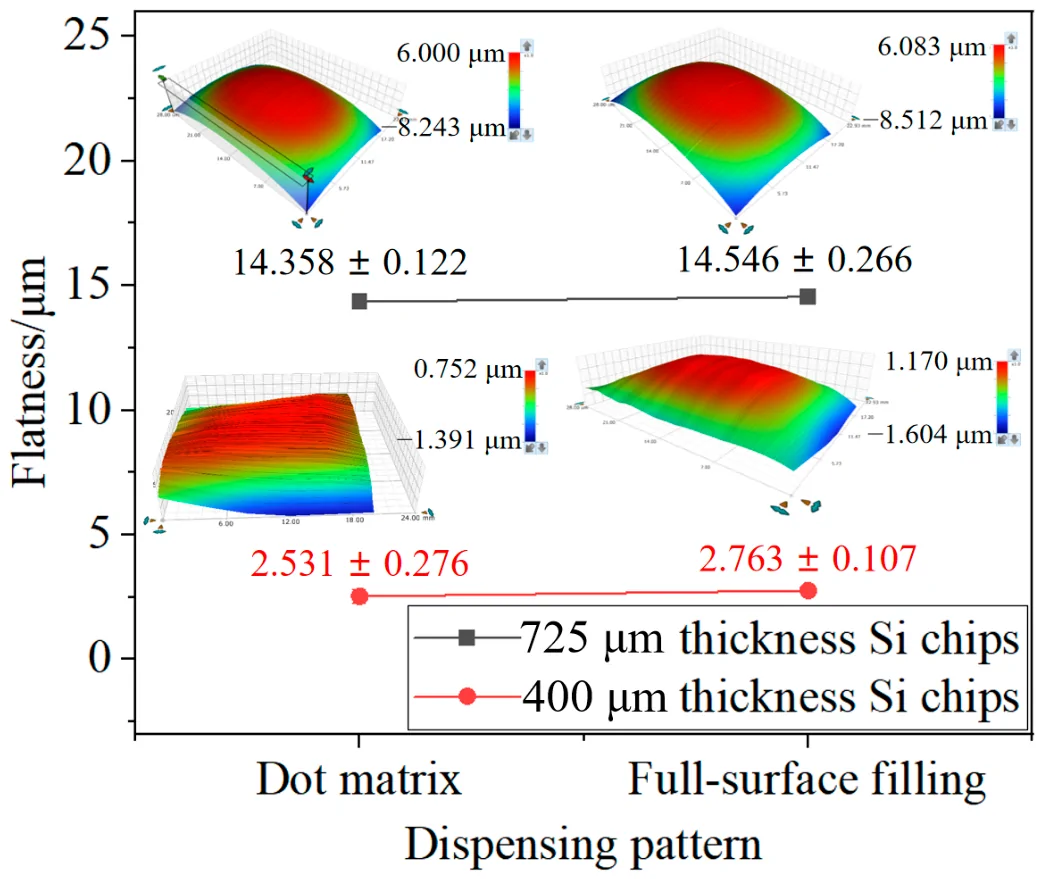
The influence of dispensing patterns on the flatness of single-chip mounting on Al_2_O_3_ substrates.

This study experimentally compares the influence of adhesive patterns (full-surface filling and dot matrix filling) on the chip-mounting flatness. Employing a same mounting process and a type ① curing curve, the 25 mm × 32 mm sized Si chip was mounted on the Al_2_O_3_ ceramic substrate, the flatness results are depicted in Figure 4. Both 400 μm and 725 μm thicknesses chips showed slight changes on the chip-mounting flatness, indicating that the variation in dispensing pattern had no significant impact on the flatness of chip mounting.

The Young’s modulus of the adhesive is considerably lower than that of the chip and the substrate, and it undergoes deformation with the deformation of the substrate during curing. In the packaging system described in this work, the substrate experiences significant shrinkage. The adhesive, which is fixed to the substrate, also shrinks in conjunction with it. Simultaneously, it applies an inward tensile force on the chip, inducing upward convex deformation of the chip. Irrespective of the adhesive’s shape, its location on the substrate remains constant, and the force it exerts on the chip does not vary significantly.

#### 3.1.4. Adhesive Layer Thickness

**Figure 5:**
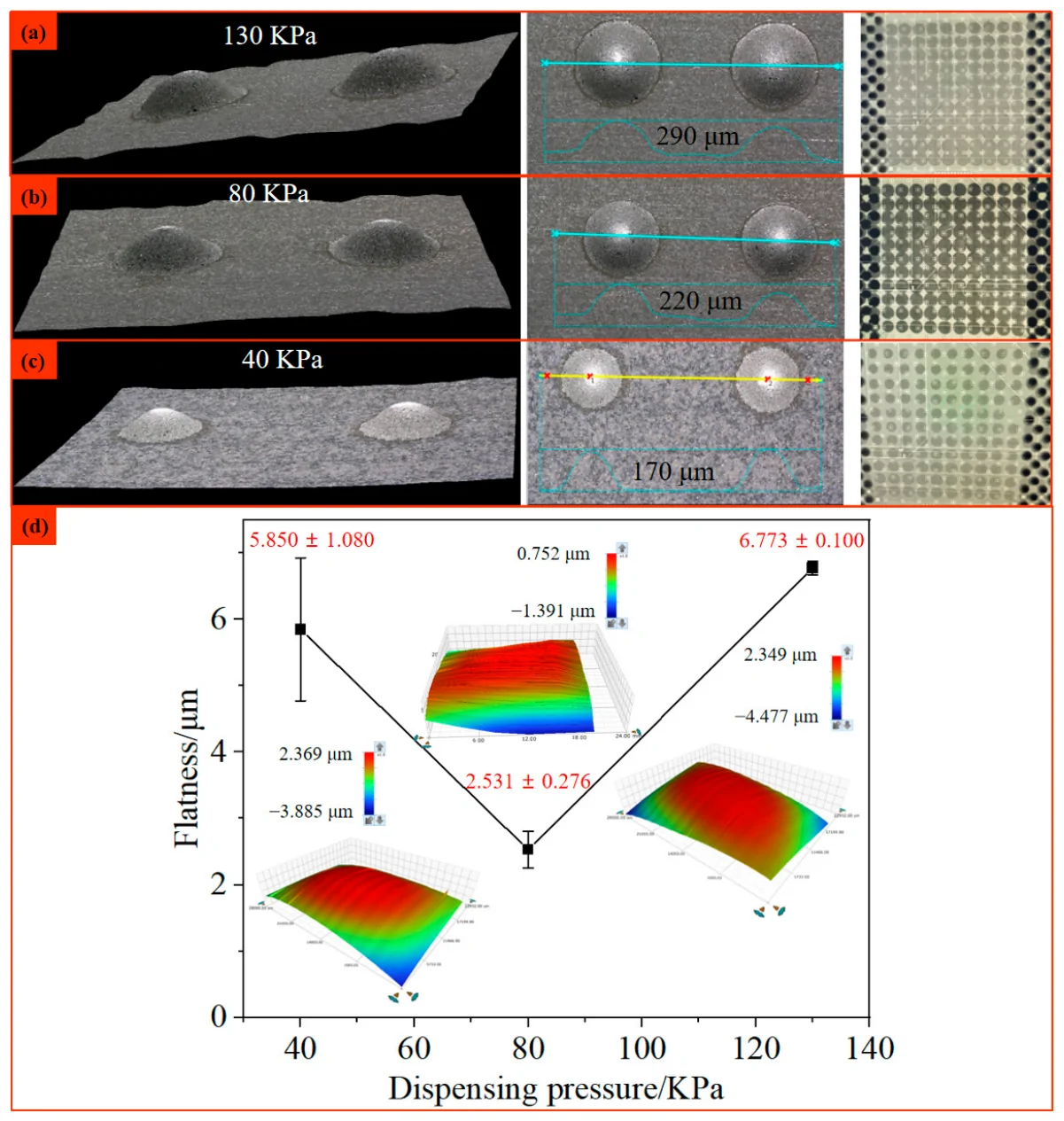
(**a**–**c**) The morphology of the adhesive dots under different dispensing pressures and the morphology of the adhesive dots after chip mounting under X-ray scanning. (**d**) The variation trend of chip flatness with the thickness of the adhesive layer.

The impact of different adhesive layer thicknesses on the focal plane flatness of the chip was assessed. Al_2_O_3_ ceramic substrates, dot matrix dispensing process, and type ① curing curve were employed. By varying the dispensing air pressure, dot matrices of differing heights were produced, resulting in varying adhesive layer thicknesses, as illustrated in Figure 5a–c. Specifically, adhesive dots dispensed at 40 KPa exhibited inadequate filling due to the limited adhesive volume. Subsequently, the flatness of the chip was assessed under varying adhesive thicknesses. The results are illustrated in Figure 5d, which suggests that adhesive thickness has minor influence on the flatness of the mounted chips. Additionally, Jiang et al. [21] utilized finite element analysis to investigate the effect of the adhesive thickness on the flatness of a 65 mm × 32 mm sized chip, with a thickness of 50 μm. Their investigation demonstrated that as the adhesive thickness rose from 25 μm to 125 μm and chip warpage increased gradually from 3.4 μm to 5 μm, further supporting the notion that adhesive thickness has a negligible effect on chip-mounting flatness.

#### 3.1.5. Curing Curve

**Figure 6:**
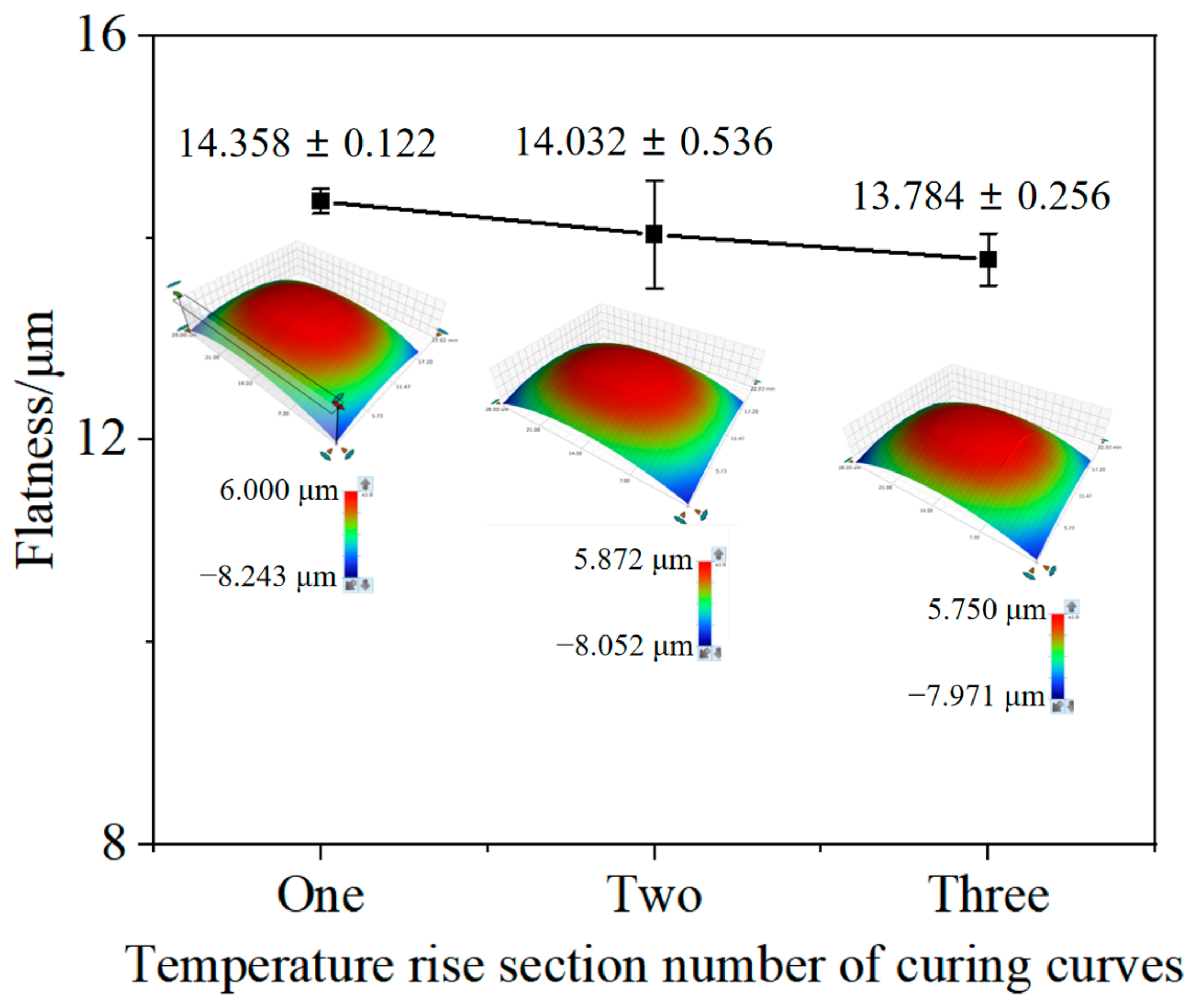
The influence of curing curves on the flatness of single-chip mounting on Al_2_O_3_ substrates.

The research on the curing process of the adhesive primarily focuses on the heating stage due to the fixed curing temperature and holding time of the adhesive. By optimizing the heating curve (e.g., slow heating or multi-step heating), researchers aim to maximize gas release and minimize internal stress accumulation [21]. This study investigated the impact of curing curves on the flatness of chip mounting using Al_2_O_3_ ceramic substrates, Si chips (32 mm × 25 mm × 725 μm), and a dot matrix dispensing pattern. The flatness of the chips cured with the three temperature curves outlined in Figure 1 was evaluated, and the results are presented in Figure 6. The findings indicate a slight decrease (14.36 µm to 13.78 µm) in chip flatness measurement results with an increasing number of temperature rise sections, suggesting that altering the temperature rise curve can only slightly enhance the flatness of the chip.

The adhesive utilized in this study necessitates a curing temperature of ≥150 °C. Curing involves a long-term physicochemical reaction. During the heating stage: when the temperature rises to 90 °C and maintains for 30 min, the adhesive’s viscosity notably diminishes, enabling it to flow easily to adequately cover and adhere to both the substrate and the backside of the chip. At 120 °C, preliminary crosslinking occurs, leading to the polymerization of small molecules into medium or large molecular chains, which facilitates partial curing between the chip and the substrate. Ultimately, upon heating to 150 °C, complete crosslinking occurs, resulting in enhanced bonding strength. It is a classic kinetic phenomenon for thermosetting resins that slower heating rates result to a lower onset curing temperature as measured by differential scanning calorimetry (DSC). In the segmented heating profile, samples remain longer in the low-temperature range, allowing adequate time for reaction accumulation. In contrast to samples rapidly heated up, significant crosslinking and curing can be triggered at a lower temperature, enabling the chip-substrate structure to achieve shape fixation at an earlier stage. During subsequent cooling to room temperature, the sample with earlier shape fixation experiences a smaller temperature difference, consequently yielding reduced warpage derived from CTE mismatch.

Based on the simulation and experimental results of the above various factors, the statistical significance of each factor was evaluated by comparing the relative change amount in the flatness of the chip as each factor is altered. And the variation in the flatness of the substrate material and chip thickness (29 μm and 23 μm, respectively) is significantly greater than that of the dispensing pattern, adhesive thickness, and curing curve (0.2 μm, 4 μm, and 0.5 μm, respectively). Therefore, it can be determined that substrate material/thickness and chip thickness are the dominant factors, while dispensing pattern, adhesive thickness, and curing curve are secondary factors.

### 3.2. High-Flatness Chip Mounting and Reliability Tests of Ultra-Large-Format Focal Plane Samples

Based on the preceding analysis of influencing factors, the substrate material and thickness, along with the chip thickness, are the primary determinants of the flatness in chip mounting. Meanwhile, in consideration of the actual application of the substrate (internal wiring), AlN is the preferred material choice of the substrate over SiC. Consequently, for the ultra-large-format focal plane imaging with dimension of 50 mm × 96 mm, the high-flatness mounting of ultra-large single chips and chip arrays were investigated by utilizing AlN ceramic substrates (with an increased thickness to 5 mm), dot matrix dispensing patterns, and type ① temperature rise curing curves.

By measuring the flatness changes in the two kinds of ultra-large-format focal plane samples before and after reliability tests, the reliability of these two ultra-large-format focal plane mounting processes was studied. Initially, the ultra-large-format focal plane samples were subjected to storage at 100 °C for 100 h, followed by the measurement of their flatness. Subsequently, these samples were subjected to 100 cycles of thermal cycling, ranging from −55 °C to 100 °C, after which their flatness was measured once more. The variations in flatness were used to evaluate the mounting reliability of the ultra-large-format focal plane samples.

#### 3.2.1. High-Flatness Mounting of an Ultra-Large Single Chip

**Figure 7:**
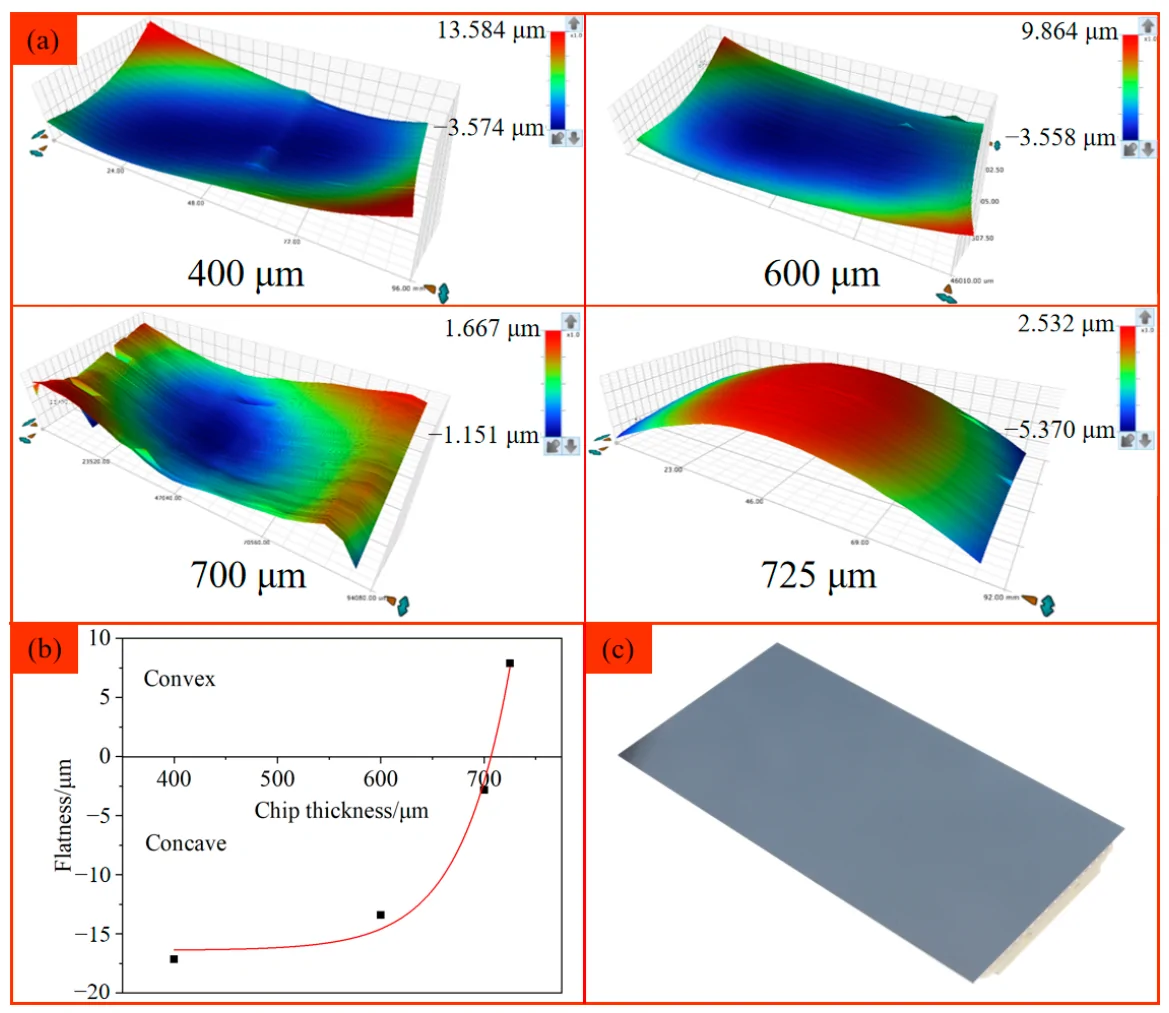
The flatness changing trend of the 50 mm × 96 mm sized chips with different thicknesses on the AlN substrate. (**a**) Flatness measuring results; (**b**) trend of flatness change; and (**c**) morphology of the ultra-large single chip post-curing.

The thickness of the chip significantly influences the flatness of chip mounting. To determine the optimal mounting flatness, the relationship between chip thickness (400 μm, 600 μm, 700 μm, 725 μm) and the flatness of an ultra-large single-chip mounting with dimensions of 50 mm × 96 mm was investigated. The results of the flatness tests are presented in Figure 7a. As chip thickness increases, the warpage decreases from a concave 17 μm to a concave 2.8 μm, ultimately transitioning to a convex 7.9 μm. This outcome demonstrates that the mounting process employed in this study can achieve micron-level flatness for ultra-large-format focal plane chips. Additionally, curve fitting was performed on the flatness variation, as illustrated in Figure 7b, revealing that the optimal chip flatness occurs at a thickness of approximately 700 μm. Both the experimental findings in Figure 3 and Figure 7 suggest that for a given chip size and substrate, there exists a theoretically optimal chip thickness that facilitates the best mounting flatness.

The initial warpage of the 400 µm and 600 µm thick chips was concave 36.899 µm and 29.768 µm, respectively (Figure S5). The flatness measurement results (Figure 7) indicated that the deformation of the three thickness chips during the curing process was 19.741 µm, 16.346 µm and 7.9 µm, respectively. This observation further corroborates the principle that thinner chip thickness results in more pronounced curing-induced deformation.

**Figure 8:**
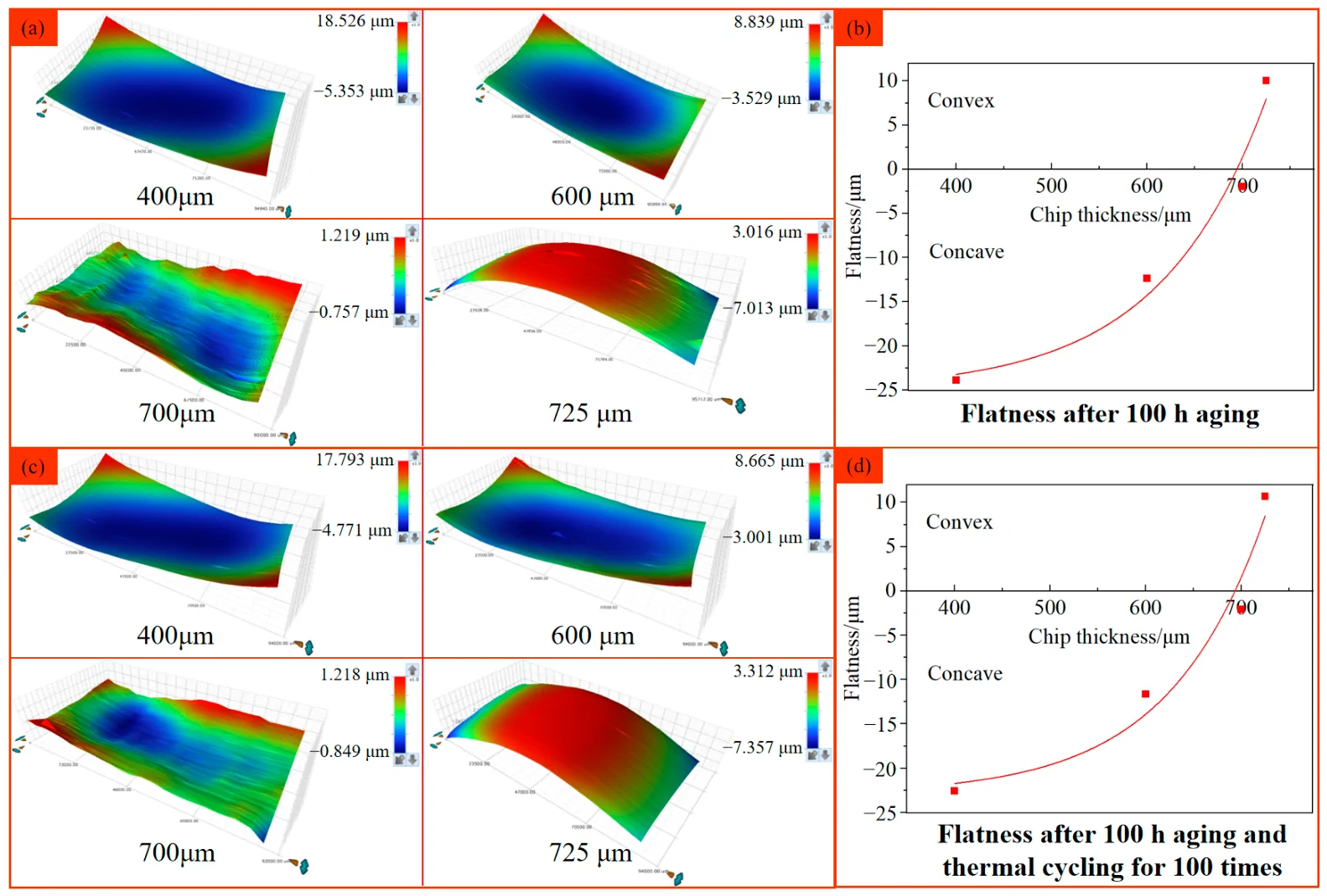
The flatness of the 50 mm × 96 mm sized chips with different thicknesses after reliability tests. (**a**) Flatness measuring results after being aged at 100 °C for 100 h; (**b**) trend of flatness change after aging; (**c**) flatness measuring results after 100 h aging and thermal cycling for 100 times; and (**d**) trend of flatness change after aging and thermal cycling.

The flatness measurement results of the ultra-large single chip following the reliability tests are presented in Figure 8. The test results indicate that for the samples with an ultra-large single chip, the flatness variation law after the reliability test with the chip thickness remains consistent with the pre-test observations. Only the flatness of the sample with a chip thickness of 400 µm increased from 17 µm to approximately 23 µm, whereas the flatness of the other samples did not change significantly. Notably, the flatness of the 700 µm thick chip remained stable at approximately 2 µm. These findings demonstrate the stability and long-term reliability of the high-flatness mounting process employed in this study.

#### 3.2.2. High-Flatness Mounting of Chip Arrays

Based on the analysis of the variation law of chip-mounting flatness, this study utilizes 25 mm × 32 mm sized Si chips, with a thickness of 400 μm, to achieve high-flatness mounting of 2 × 3 image sensor chip arrays. During the curing process, the chips in the unit do not affect each other, thereby ensuring the flatness of each individual piece in the array. However, during the actual mounting process of the chip array, variations in factors such as the flatness of the splicing substrate, the uniformity of dispensing, and the consistency of mounting pressure lead to differences in the warpage direction and relative height among individual pieces within the splicing unit, resulting in an excessive flatness of the splicing unit. The consistency of equipment and raw materials has emerged as the primary factor affecting the flatness of the splicing unit.

**Figure 9:**
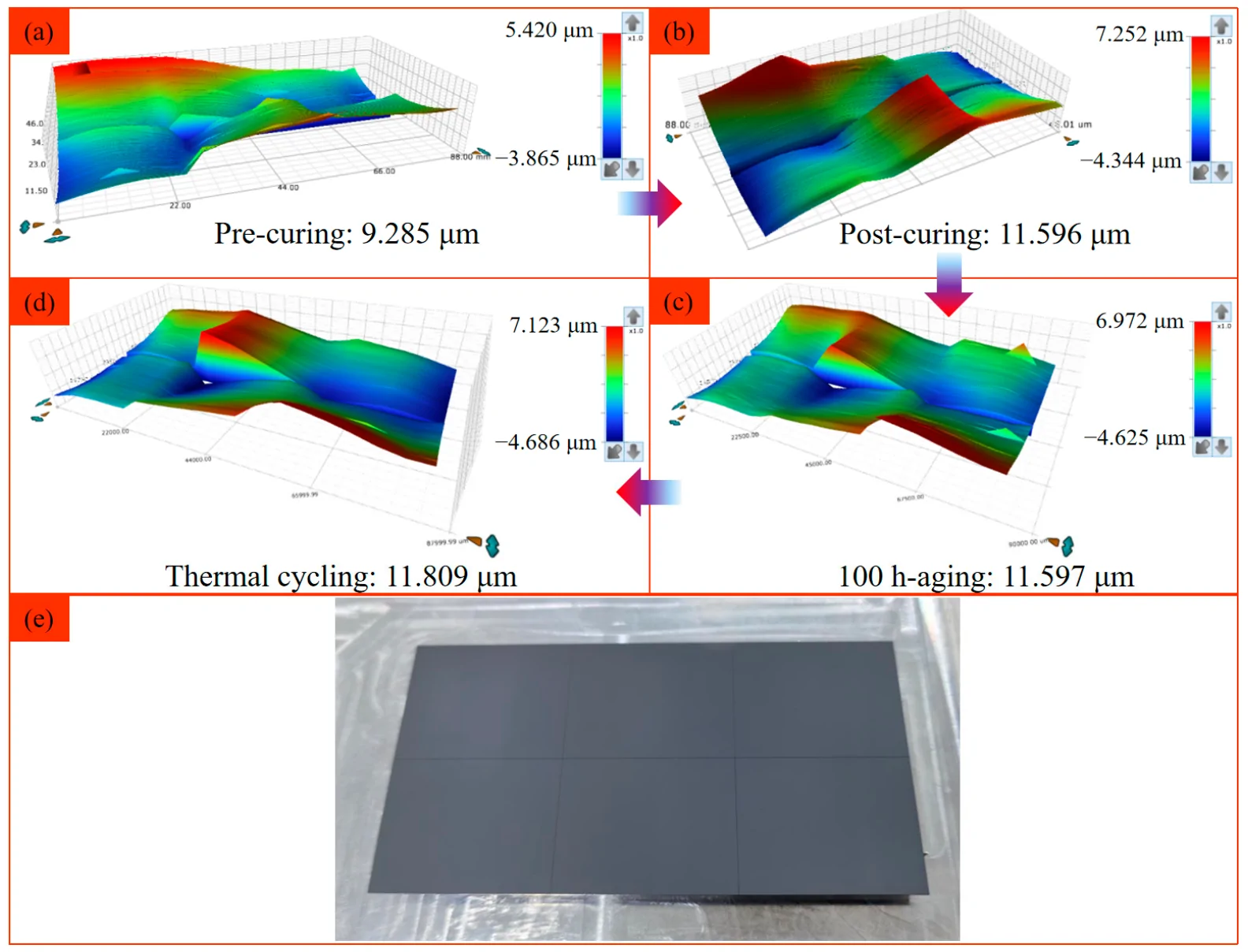
Flatness of the 2 × 3 image sensor chip array and its reliability test results. (**a**) The flatness of the chip array prior to curing; (**b**) the flatness of the chip array post-curing; (**c**) the flatness of the chip array after being aged at 100 °C for 100 h; (**d**) the flatness of the chip array after 100 h of aging and thermal cycling for 100 times; and (**e**) morphology of the 2 × 3 image sensor chip array.

By adopting the fine-tuning method after mounting the chip array, this study successfully achieved high-flatness mounting of the splicing unit (Figure 9). To demonstrate the validity and reproducibility of this fine-tuning method, the measurement results of another two samples are presented in the supporting information (Figure S6). Figure 9a,b presents the flatness measuring results for the 2 × 3 image sensor splicing unit pre- and post-curing. The initial flatness of the splicing unit pre-curing was measured at 9.2 μm, while the overall flatness after curing was recorded at 11.6 μm. The flatness measurement results of the 2 × 3 chip arrays post the reliability tests are presented in Figure 9c,d and Figure S6. No significant alterations in flatness were observed across the three samples. For instance, the flatness of the sample in Figure 9 only changed from 11.6 µm to 11.8 µm after testing. In conclusion, the chip array splicing method can also achieve micron-level flatness mounting with ultra-large-format focal plane.

### 3.3. Discussion

In conclusion, this research has developed a high-flatness mounting methods for packaging structure of image sensor chip-adhesive-substrate, regardless of its focal plane size. Through the coordinated optimization of the dominant factors (packaging materials, structural dimensions) and secondary factors (mounting processes), high-flatness mounting of image sensor chips can be achieved. Initially, the packaging structure is optimized by considering the effects of substrate material and chip thickness on the flatness of chip mounting. Subsequently, the flatness is further refined through the careful selection of the mounting process.

This method has been verified on a 50 mm × 96 mm sized ultra-large-format focal plane splicing units. By optimizing the selection of substrate materials and chip thickness, high-flatness mounting (2.8 μm) was achieved. And the reliability tests further proved the long-term reliability of this method. Compared with the mounting flatness of ultra-large-format focal plane splicing units both domestically and internationally, as shown in Table 1, in terms of both size and flatness, it has reached the international advanced level.

## 4. Conclusions

This work conducted research on the high-flatness mounting process of image sensor chips for 50 mm × 96 mm sized ultra-large-format focal plane splicing units. Through the coordinated optimization of packaging materials, structural dimensions, and mounting processes, the high-flatness mounting can be achieved for image sensor chips, irrespective of its focal plane size. It is concluded that the dispensing pattern, adhesive layer thickness and curing curve are the secondary factors affecting the flatness of chip mounting, while the substrate material, thickness, chip thickness are the primary factors. Based on the influence law of chip thickness on the chip mounting warpage, it can be determined that for substrates and chips of specific sizes, there exists an optimal chip thickness that yields the minimum chip-mounting flatness. High-flatness mounting of 50 mm × 96 mm sized single-chip or 2 × 3 chip splicing arrays has been achieved. Specifically, the flatness of the large-sized single chip attains 2.8 μm, and the flatness of the 2 × 3 chip splicing array reaches 11.6 μm. Furthermore, following high-temperature storage and thermal cycling tests, the flatness of these ultra-large-format focal plane samples did not significantly deteriorate, demonstrating the high reliability of the chip mounting process employed in this study.

## Figures and Tables

**Table 1:** Focal plane flatness specifications for the splicing sensor chips in large-scale optical survey telescopes worldwide [13].

Survey Telescopes	CCD Types	CCD Numbers	Dimension of the CCDs	Nominal PV of the CCDs	PV of the Overall Focal Plane
Pan-STARRS [14,15]	CCID58	60	50 mm × 50 mm	10 μm	40 μm
Subaru/HSC [16]	CCID-20	116	30.72 mm × 61.44 mm	20 μm	34 μm
SkyMapper	CCD44-82	32	30.72 mm × 61.44 mm	20 μm	64 μm
J-PAS/JPCam [17]	CCD290-99	14	92.2 mm × 92.4 mm	15 μm	27 μm
LSST [18]	CCD250-82	189	42 mm × 42 mm	7 μm	22 μm
WFST [19]	CCD290-99	9	92.2 mm × 92.4 mm	15 μm	20 μm
HFMC-Cam [20]	GSENSE6060	25	61 mm × 61 mm	<10 μm	<20 μm
/	[21]	/	62 mm × 32 mm	<10 μm	/

**Table 2:** Materials and the parameters.

Materials	CTE (10^−6^·°C^−1^)	Young’s Modulus (Gpa)	Poisson Ratio	Thermal Conductivity (W/m·K)
Al_2_O_3_	6.7	350	0.1	25
AlN	4.4	320	0.23	200
SiC	2	450	0.14	180
Si	2.6	187	0.2	150
Adhesive	33	10	0.38	1

## Data Availability

The data that has been used is confidential.

## Supplementary Materials

The following supporting information can be downloaded at: https://www.mdpi.com/article/10.3390/s26216783/s1, Figure S1: The morphology of adhesive dots in different states under X-ray scanning; Figure S2: The clarity of four spliced chips in the same field of view; Figure S3: The warping process of the chip during curing process; Figure S4: The warpage of the chips (25 mm × 32 mm) post-thinning and cutting. (a) 200 µm thickness chip; (b) 400 µm thickness chip; Figure S5: The warpage of the chips (50 mm × 96 mm) post-thinning and cutting. (a) 400 µm thickness chip; (b) 600 µm thickness chip; Figure S6. The flatness measurement results of three 2 × 3 chip arrays samples before and after reliability tests. (a) sample 1; (b) sample 2; (c) sample 3.
