# Supplementary material for: The High-Flatness Mounting Process of Image Sensor Splicing Units with Ultra-Large Format Focal Plane
Source: Sensors (Basel). 2026 Oct 27;26(21):6783. doi: 10.3390/s26216783 (PMC13766110; doi:10.3390/s26216783)
Supplement: Supplementary file 1 [file sensors-26-06783-s001.zip › sensors-4638096-supplementary.pdf]

Supporting Information

The high flatness mounting process of image sensor splicing units with ultra-large format focal plane

Hongjian Li, Linxiao Jing, Lipeng Jing, Haoran Li, Qijun Zhou, Yongchao Wu*

1. High flatness fine-tuning methods for chip arrays

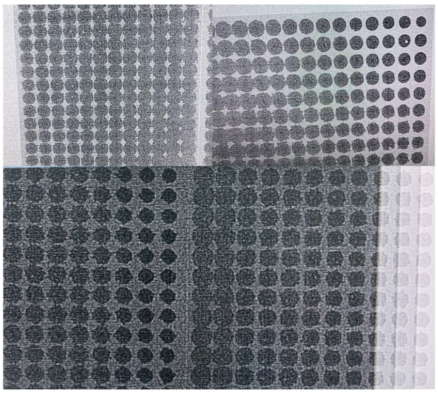

Figure S1. The morphology of adhesive dots in different states under X-ray scanning.

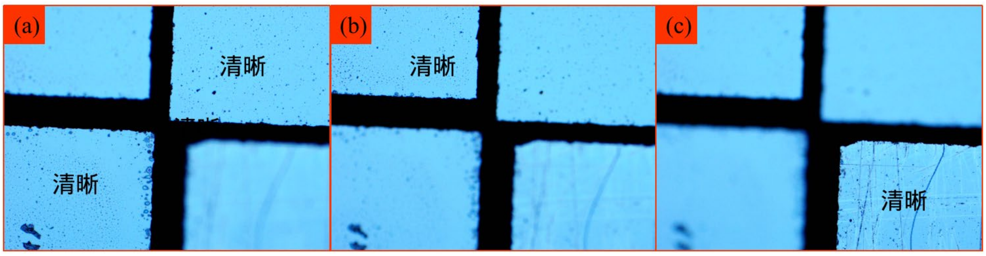

Figure S2. The clarity of four spliced chips in the same field of view.

2. The variation law of the chip mounting flatness

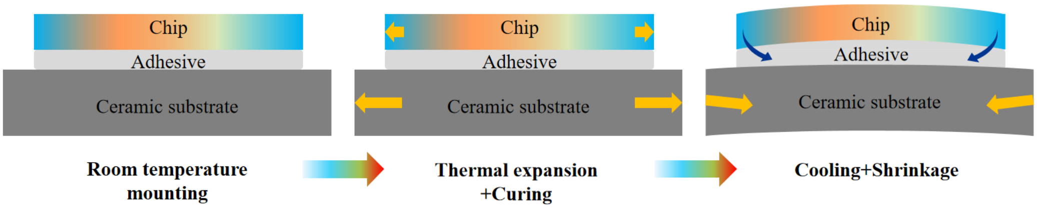

Figure S3. The warping process of the chip during curing process.

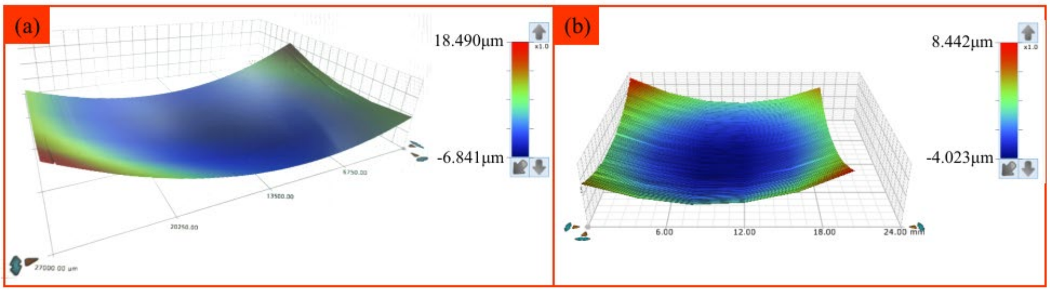

Figure S4. The warpage of the chips (25mm×32mm) post-thinning and cutting. (a) 200μm thickness chip; (b) 400μm thickness chip.

3. High Flatness chip mounting and reliability tests of ultra-large-format focal plane samples

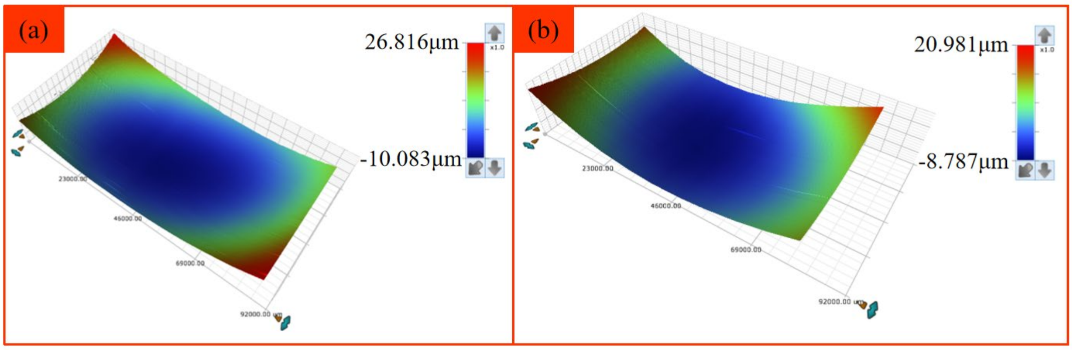

Figure S5. The warpage of the chips (50mm×96mm) post-thinning and cutting. (a) 400μm thickness chip; (b) 600μm thickness chip.

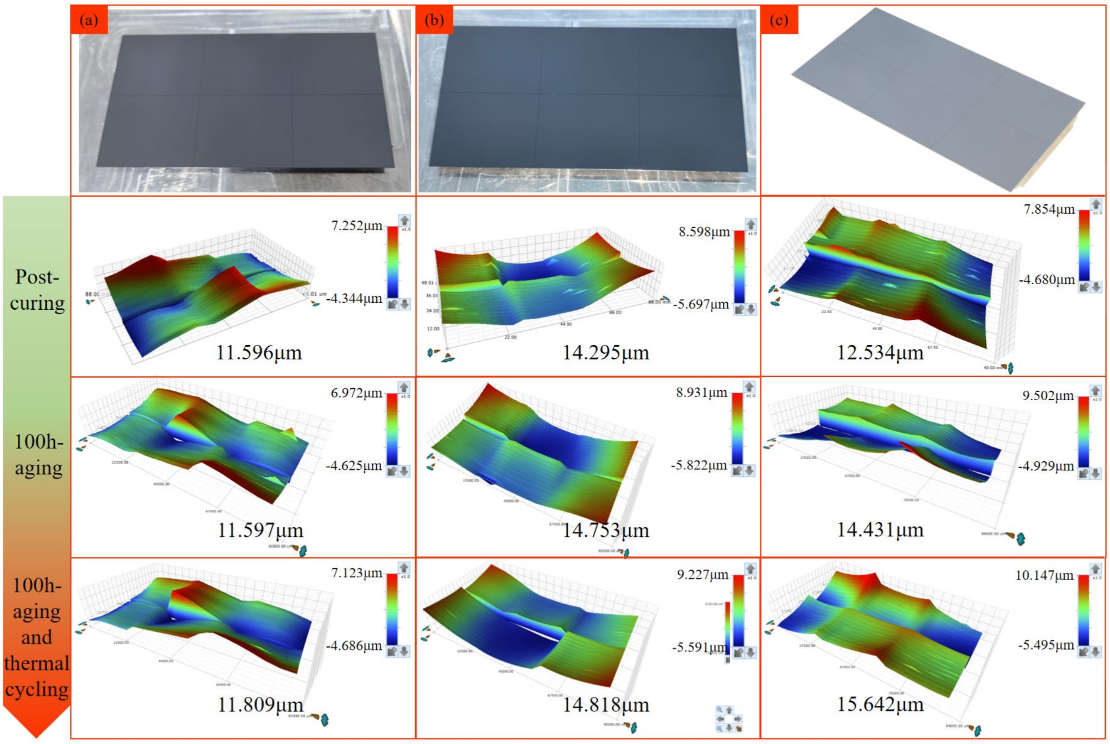

Figure S6. The flatness measurement results of three 2×3 chip arrays samples before and after reliability tests. (a) sample 1; (b) sample 2; (c) sample 3.
